# A Novel Homozygous Mutation in the MYO5B Gene Associated With Normal-Gamma-Glutamyl Transferase Progressive Familial Intrahepatic Cholestasis

**DOI:** 10.7759/cureus.19326

**Published:** 2021-11-07

**Authors:** Nihal Uyar Aksu, Orhan Görükmez, Özlem Görükmez, Ayşen Uncuoğlu

**Affiliations:** 1 Pediatric Gastroenterology, Health Sciences University Derince Training and Research Hospital, Kocaeli, TUR; 2 Genetics, Bursa State Hospital, Bursa, TUR; 3 Pediatric Gastroenterology, Kocaeli University, Kocaeli, TUR

**Keywords:** novel mutation, genetic mutation, normal/low ggt, progressive familial intrahepatic cholestasis, myo5b

## Abstract

The genetic defect of MYO5B is usually associated with microvillus inclusion disease (MVID). MYO5B mutations are one of the rare causes of progressive familial intrahepatic cholestasis (PFIC) with normal/low gamma-glutamyl transferase (GGT). In this report, we discuss the case of a nine-month-old girl with low-GGT cholestasis whose next-generation sequencing (NGS) showed a homozygous splicing variation (c.3045+3A>T) on the MYO5B (NM_001080467) gene, which was a novel mutation. We identified that this mutation had a disease-causing effect in silico analysis.

## Introduction

Progressive familial intrahepatic cholestasis (PFIC) is a group of autosomal recessive cholestatic disorders caused by the defects of the biliary canalicular transport. They usually appear in the first month or the first year of life. The estimated incidence of PFIC varies between 1/50,000 and 1/100,000. They used to be classified into three groups: PFIC1 (Byler disease), PFIC2 [bile salt exporter pump (BSEP) deficiency], and PFIC3 [multi drug resistance 3 (MDR3) deficiency], but recently more comprehensive genetical studies have revealed new genetic mutations. One of these mutations was identified in the gene MYO5B, which encodes the protein myosin 5b (MYO5B). MYO5B participates in plasma membrane recycling, transcytosis, and epithelial cell polarization [1,2]. It is found in various tissues like enterocytes, hepatocytes, kidneys, and respiratory epithelial cells. The most well-known genetic defect of MYO5B is seen in enterocytes, which causes microvillus inclusion disease (MVID). MVID is a congenital, autosomal recessive disorder characterized by intractable diarrhea requiring parenteral nutrition and intestinal transplantation. Low-gamma-glutamyl transferase (GGT) cholestasis was observed in some MVID patients before or after liver transplantation. This low-GGT cholestasis could not have been attributed to parenteral nutrition or anticalcineurin inhibitors used after intestinal transplantation [3,4]. Besides, it has been reported that genetic mutations in MYO5B can cause isolated cholestasis. These patients present with low-GGT cholestasis, pruritus, and hepatomegaly in the first year of life [5]. 

In hepatocytes, MYO5B binds to GTPase RAB protein, RAB11A, to target adenosine triphosphate (ATP)-binding-cassette (ABC) transporters like BSEP to the canalicular membrane [6]. This report presents a patient with low-GGT cholestasis whose genetic analysis revealed a novel mutation in MYO5B.

Written informed consent from the patient’s parents was taken to use and publish all the data and pictures used in this report.

## Case presentation

**Figure 1:**
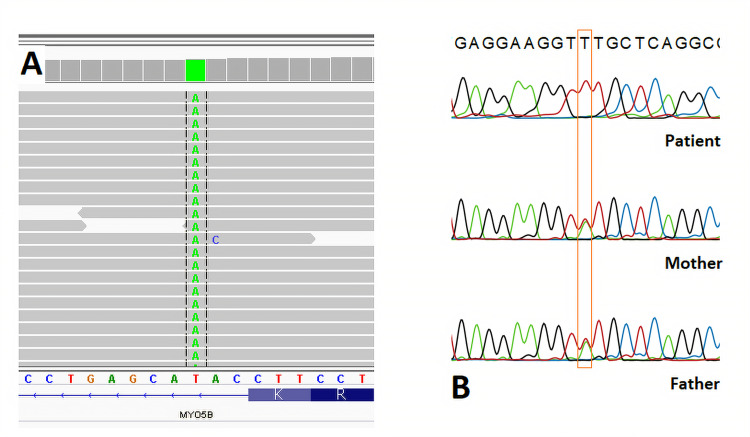
Molecular genetic analysis of the family A. Excerpt of next-generation sequencing data visualized using Integrative Genomics Viewer (IGV). The black frame indicates the mutation (chr18:47421308T>A) Note: The mutation is shown as a T→A change because IGV always displays the forward strand, and in the MYO5B gene, the coding strand is the reverse one [7] B. Results of DNA sequencing. The splicing germline mutation, c.3045+3A>T on the MYO5B (NM_001080467) gene of the family (indicated by orange frame)

A nine-month-old girl presented with jaundice, pruritus, and rickets. Her jaundice and pruritus had started within the previous month. She had been diagnosed with rickets before she was referred to our department for cholestasis. She was on vitamin D and calcium replacement therapy. She had been born to consanguineous parents and had a healthy elder sister. Her prenatal history was unremarkable. She had been born at term with a birth weight of 2,500 g. There was no family history of chronic liver or bowel disease. Her weight and height were 6.3 kg (<3rd percentile, -2.47 standard deviation score) and 63 cm (<3rd percentile, -3 standard deviation score) respectively. Her weight for length was normal. She had scleral icterus, scars on her arms due to pruritus, and mild hepatomegaly. She did not have discolored stools or diarrhea. Abdominal ultrasonography was consistent with an enlarged liver with heterogeneous parenchyma and a normal biliary tree. Her laboratory tests showed low GGT activity and mildly elevated transaminase and bilirubin levels (Table 1). Her serum bile acid levels were elevated (135.23 mmol/L, normal range: <10 mmol/L). There was no exocrine pancreatic insufficiency. Fecal elastase level was >500 mg/g. Her laboratory tests evaluating tubulopathy and urine organic acid analysis were normal. The prothrombin time was 23.9 seconds, which returned to normal after vitamin K replacement. The level of vitamin D was <7 ng/ml. She was already taking vitamin D as prescribed by the Pediatric Endocrinology Department. Vitamins A, E, K, and ursodeoxycholic acid (UDCA) supplementation were started. The etiology of low-GGT cholestasis was investigated genetically. No liver biopsy was performed. Next-generation and Sanger sequencing were used for the patient. The Clinical Exome Solution (Sophia Genetics SA, Saint-Sulpice, Switzerland) was performed on NextSeq 500 system (Illumina, San Diego, CA) for exome enrichment, with all procedures carried out according to the manufacturer’s protocols. All bioinformatics analyses, variant filtering, and interpretations were performed using the Sophia DDM™ platform (Sophia Genetics SA). Next-generation sequencing (NGS) showed a homozygous splicing variation (c.3045+3A>T) on the MYO5B (NM_001080467) gene (Figure 1A). Segregation analysis was performed by the Sanger sequencing method (Figure 1B).

As the patient resided in a city far away from our clinic, she was only able to come for examination at the age of 28 months. Her parents reported that the patient had started to have diarrhea, and her pruritus had stopped at the age of 16 months. She had one to three instances of watery stools per day. She weighed 9 kg (<3rd percentile, -2.76 standard deviation score) and her height was 73 cm (<3rd percentile, -4.64 standard deviation score). Her body mass index was 16.9 kg/m^2^ (77th percentile), and she had hepatomegaly. Her bilirubin levels were normal (Table 1). Laboratory test results for infectious causes and celiac disease were also normal. Supportive treatment was provided for diarrhea. She was evaluated for renal Fanconi syndrome as patients carrying MYO5B mutations could develop this syndrome [8]. However, there was no sign of the syndrome. At her last visit, she was still on UDCA therapy, and it was decided that she did not need to use any other antipruritic drug.

**Table 1:** Laboratory results of the patient at 9th and 28th months *These are the normal values of our laboratory AST: aspartate aminotransferase; ALT: alanine aminotransferase

Variables	9th month	28th month	Normal value*
GGT (U/L)	11	12	6-42
AST (U/L)	61	33.2	<32
ALT (U/L)	49.7	35.3	<33
Total bilirubin (mg/dl)	3.85	0.31	<1.2
Direct bilirubin (mg/dl)	2.25	0.07	<0.3
Albumin (g/dl)	4.18	4.3	3.97-4.94
Prothrombin time (seconds)	23.9	16.8	11.5-15.5

## Discussion

MYO5B mutations causing MVID were first identified by Müller et al. [9] in 2008. Normal/low-GGT cholestasis accompanying MVID has been reported as an atypical finding or a consequence of parenteral nutrition before or after intestinal transplantation [10-12].

Girard et al. [13] investigated the etiology of low-GGT cholestatic liver disease among their patients with MVID. In their study, there were 28 children with MVID, and eight among them developed intrahepatic cholestasis either early in life or after intestinal transplantation. The mechanisms of cholestasis were explained by the impairment of the MYO5B/RAB11A apical recycling endosome pathway in hepatocytes, changed expression of BSEP at the canalicular membrane, and increased ileal bile acid absorption and hepatic bile acid uptake.

Later, in 2017, Gonzales et al. [5] reported that MYO5B mutations could cause cholestasis with normal serum GGT activity in children without MVID. Immunostaining data of their patients supported the hypothesis that MYO5B mutations caused an impairment of MYO5B/RAB11A interaction, altering the targeting of ABC transporters (i.e., BSEP, MDR3) to the canalicular membrane of the hepatocytes, and thus impaired the canalicular bile secretion [5,13,14]. Qui et al. [15] researched the underlying genetic mutations in patients with normal-GGT cholestasis with unknown etiology. Whole exome sequencing and targeted sequencing were performed in their study. Mutations in MYO5B were found to be around 20% among the patients with normal-GGT cholestasis with unknown etiology. These patients had no diarrhea, and isolated cholestasis was thought to be caused by non-severe mutations in MYO5B.

Gonzales et al. [5] reported five patients presenting with jaundice, pruritus, and discolored stools, who had cholestasis resembling classical normal-GGT PFIC. The starting age of their symptoms was around one year. Similarly, our patient presented with jaundice and pruritus at the age of nine months. This could be a distinguishing feature for MYO5B-cholestasis as PFIC1 and PFIC2 are usually present in the first months of life [16,17].

In a recently published series involving six patients, mutations in MYO5B associated with early-onset cholestasis were recorded [18]. These patients had severe pruritus. Some patients needed a wide selection of antipruritic medication, whereas others required early surgical intervention. Our patient had adequate relief with UDCA and did not need any further medication.

Our patient started to have mild diarrhea when she was 17 months old. She had one to three watery stools during the day. Gonzales et al. [5] had one patient who had several episodes of severe acute diarrhea. As the duodenal histology was normal, it was suggested to be a coincidence. Qui et al. [15] have mentioned that one of their patients had a history of loose stools until the age of three. Cockar et al. [18] have reported minimal incidence of gastrointestinal disease in their series. Four of six patients had no evidence of gastrointestinal disease, whereas two patients had intractable diarrhea for a while. An upper gastrointestinal system endoscopy has not been performed for our patient, and hence we cannot comment on the duodenal histology and the relationship of this symptom with MVID.

In summary, NGS of our patient showed a homozygous splicing variation (c.3045+3A>T) on the MYO5B (NM_001080467) gene. The variant has not been previously reported in the Human Gene Mutation Database (HGMD) [19] and population studies (ExAC: Exome Aggregation Consortium and 1000 Genomes Project). In silico analysis, programs such as Human Splicing Finder (HSF) [20] and MutationTaster [21] showed that the change might have pathogenic effects. The fact that the change was not reported in the population, the parents were carriers in the segregation analysis, and the interpretation of the alteration of the WT Donor site as most probably affecting splicing according to HSF showed us that this variant could be the cause of the disease (Table 2).

**Table 2:** Interpretation of the variant according to HSF Interpretation: alteration of the wild type donor site, most probably affecting splicing HSF: Human Splicing Finder; REF: reference; ALT: alteration

Algorithm/matrix	Position	Sequences	Variation
HSF donor site (matrix GT)	Chr18:49894943	-REF: AAGGTATGC -ALT: AAGGTTTGC	89.74 > 77.96 → -13.13%
MaxEnt donor site	Chr18:49894943	-REF: AAGGTATGC -ALT: AAGGTTTGC	9.55 > 5.42 → -43.25%

Until now, different types of mutations have been reported in the MYO5B gene, albeit in a small number. In a functional study by Overeem et al. [22] about the MYO5B gene, the authors specifically observed that missense changes caused cholestasis, and changes that constituted a premature stop codon caused MVID. In another study conducted by van IJzendoorn et al. [23] on 22 cases with MYO5B mutation, the authors emphasized that it was difficult to make a general comment on the disease and that personalized approaches would be more appropriate.

## Conclusions

Based on our findings, the effects of MYO5B mutations, which are heterogeneous in terms of clinical findings, will become more apparent with the increase in the number of cases and functional studies. The clinicians should be aware of MYO5B mutations in patients with isolated cholestasis. We believe that this novel MYO5B mutation should be investigated in patients who have the characteristics of low-GGT PFIC.
